# 519 Comparing Evidence Based Equations and Indirect Calorimetry in Moderate Size Burns

**DOI:** 10.1093/jbcr/irae036.154

**Published:** 2024-04-17

**Authors:** Lacey Patton, Scott W Mueller, Arek J Wiktor, Cameron Gibson

**Affiliations:** University of Colorado Hospital, Littleton, CO; UCHealth and University of Colorado School of Pharmacy, Aurora, CO; University of Colorado Hospital, Aurora, CO; University of Colorado, Aurora, CO; University of Colorado Hospital, Littleton, CO; UCHealth and University of Colorado School of Pharmacy, Aurora, CO; University of Colorado Hospital, Aurora, CO; University of Colorado, Aurora, CO; University of Colorado Hospital, Littleton, CO; UCHealth and University of Colorado School of Pharmacy, Aurora, CO; University of Colorado Hospital, Aurora, CO; University of Colorado, Aurora, CO; University of Colorado Hospital, Littleton, CO; UCHealth and University of Colorado School of Pharmacy, Aurora, CO; University of Colorado Hospital, Aurora, CO; University of Colorado, Aurora, CO

## Abstract

**Introduction:**

Burn survivors require additional calories due to hypermetabolic demands for large burn injuries consisting of >20% total body surface area (TBSA). Evidence-based energy equations for the burn population can significantly overestimate or underestimate caloric needs. There is limited research regarding metabolic demands of moderate size burns, consisting of 10-19.9% TBSA. We aimed to correlate patient specific indirect calorimetry (IC) results with expected caloric needs using equation estimates.

**Methods:**

This was a single center retrospective cohort study at an American Burn Association (ABA) verified burn center. We included patients with 10% and greater TBSA between 3/15/23 and 9/1/23 with a validated IC. Resting energy expenditure (REE) multiplied by 1.3 activity factor (IC 1.3) were compared to the Xie and Carlson, if within the first 30 days post injury, or Milner, if after 30 days since injury. In addition, we compared the Mifflin St Jeor (MSJ) equation to REE (no activity factor) in patient with TBSA 10-19.9%. Correlations coefficients and matched mean differences to assess for bias of equation based versus IC based caloric needs.

**Results:**

A total of 15 patients, with a median age and TBSA of 31 years (30, 52.5) and 20% (13.6, 33.1), had 39 IC assessments performed. The average (standard deviation) of caloric requirements for IC 1.3, Xie, and Carlson/Milner were 3112 (±541), 2857 (±707), and 2881 (±753) Kcal, respectively. Caloric requirements by IC 1.3 correlated weakly with Xie (0.36, p=0.024) and Carlson/Milner (0.31, p=0.055). Xie and Carlson/Milner correlated strongly (0.94, p< 0.001). Xie significantly underpredicted caloric needs while the Carlson/Milner trended towards a negative bias compared to IC 1.3 (matched mean difference: -255, p=0.033 and -231, p=0.072). In those 10-19.9% TBSA, 7 patients (median 12.75%; 10.8, 14.75) had 11 assessments. Average REE by IC was 2270 (±285) vs 1517 (±167) Kcal by MSJ. There was a moderate correlation between IC REE and MSJ (0.65, p=0.029). However, MSJ was significantly negatively biased compared to IC REE (matched mean difference -753, p< 0.011).

**Conclusions:**

Xie and Carlson/Milner equations had a weak correlation with IC 1.3 estimated caloric requirements. These equations tended to underpredict actual caloric needs. In the 10-19.9% TBSA group, MSJ significantly underpredicted IC REE. This suggests patients with TBSA 10-19.9% are hypermetabolic and may require more intense nutrition support. Larger studies in the TBSA range are required to validate these results.

**Applicability of Research to Practice:**

Evidence-based energy equations are correlated weakly with patient-specific IC based results and should only be used if IC is unable to be performed. Greater caloric provision may be needed in the 10-19.9% TBSA burn population.

